# Arbitrary polarization conversion dichroism metasurfaces for all-in-one full Poincaré sphere polarizers

**DOI:** 10.1038/s41377-021-00468-y

**Published:** 2021-01-27

**Authors:** Shuai Wang, Zi-Lan Deng, Yujie Wang, Qingbin Zhou, Xiaolei Wang, Yaoyu Cao, Bai-Ou Guan, Shumin Xiao, Xiangping Li

**Affiliations:** 1 Guangdong Provincial Key Laboratory of Optical Fiber Sensing and Communications, Institute of Photonics Technology, Jinan University, 510632 Guangzhou, China; 2 Ministry of Industry and Information Technology Key Lab of Micro-Nano Optoelectronic Information System, Harbin Institute of Technology, 518055 Shenzhen, China; 3 Institute of Modern Optics, Nankai University, 300350 Tianjin, China

**Keywords:** Metamaterials, Photonic devices

## Abstract

The control of polarization, an essential property of light, is of broad scientific and technological interest. Polarizers are indispensable optical elements for direct polarization generation. However, arbitrary polarization generation, except that of common linear and circular polarization, relies heavily on bulky optical components such as cascading linear polarizers and waveplates. Here, we present an effective strategy for designing all-in-one full Poincaré sphere polarizers based on perfect arbitrary polarization conversion dichroism and implement it in a monolayer all-dielectric metasurface. This strategy allows preferential transmission and conversion of one polarization state located at an arbitrary position on the Poincaré sphere to its handedness-flipped state while completely blocking its orthogonal state. In contrast to previous methods that were limited to only linear or circular polarization, our method manifests perfect dichroism of nearly 100% in theory and greater than 90% experimentally for arbitrary polarization states. By leveraging this attractive dichroism, our demonstration of the generation of polarization beams located at an arbitrary position on a Poincaré sphere directly from unpolarized light can substantially extend the scope of meta-optics and dramatically promote state-of-the-art nanophotonic devices.

## Introduction

Polarization control is essential for tailoring light–matter interactions and is the foundation for many applications, such as polarization imaging^1,2^, nonlinear optics^3,4^, data storage^5,6^, and information multiplexing^7–9^. A linear polarizer, which is a polarization optical element that filters a specific linear polarization from unpolarized light, plays an important role in both polarization generation and manipulation. However, the generation of arbitrary polarization states other than linear polarization usually requires cascading of multiple optical polarization elements, including both linear polarizers and waveplates based on anisotropic materials^10^ or nanostructures^11^, leading to bulky optical systems that are far from the long-sought miniaturization and integration. Recently, circular polarizers that can directly generate circular polarization from unpolarized light by exploiting the extremely large optical chiral response of 3D chiral nanostructures were proposed^12–15^. To miniaturize these devices, structures with planar optical chirality with only in-plane chiral geometries and preservation of the mirror symmetry in the light propagation direction were proposed^16^, with the advantages of easy fabrication and on-chip integration. A variety of planar chiral structures, including fish-scale^16^, asymmetric split ring^17^, gammadion^18^, L-shaped^19^, and Z-shaped^20^ structures, have been investigated. In these structures, exotic phenomena such as circular conversion dichroism (CCD) and asymmetric transmission (AT) occur, in which only one kind of circularly polarized light can be transmitted and converted to its handedness-flipped state, while the orthogonal circular polarization is completely blocked^19–21^.

In parallel, tremendous progress has been made in the area of metasurfaces composed of artificial meta-atoms with tailored phase responses^22–36^, leading to lightweight optical devices such as metalenses^37–39^ and meta-holograms^22,40^. Polarization control by metasurfaces typically involves anisotropic meta-atom designs to impose different phase retardations on orthogonal linear polarizations, analogous to optical birefringence. By combining the anisotropic dynamic phase with the geometric phase, independent phase retardation can be imposed on any pair of orthogonal polarization states^41^. Elliptical birefringence has also been realized with independent phase manipulation based on elliptical eigenpolarization states, leading to arbitrary polarization conversion under a specified input polarization^42^. In addition, combining the geometric phase with the detour phase can allow simultaneous control of the spatially varying arbitrary polarization and phase profiles, leading to powerful vectorial hologram applications^29,34,43^. However, all of these metasurface polarization optics require an additional polarizer to generate incident beams with well-defined polarization, precluding their applicability in monolithic polarization generators working directly with unpolarized light.

In this paper, we propose an effective approach to achieve full Poincaré sphere polarizers in one step by extending CCD to arbitrary polarization conversion dichroism (APCD) by means of a monolayer metasurface. By using dimerized metamolecules^29,34,44,45^ composed of a pair of birefringent meta-atoms with properly tailored anisotropic phase responses and relative orientation angles, the collective interference of far-field radiation from those meta-atoms can be controlled to generate APCD. This system is able to preferentially transmit one polarization state that can be located at an arbitrary position on a Poincaré sphere^46^ and convert it into transmitted light with flipped handedness while completely blocking the orthogonal polarization state. This APCD metasurface is capable of generating an arbitrarily polarized beam located at an arbitrary position on the Poincaré sphere, irrespective of input polarization, and thus acts as a polarizer that can cover the full Poincaré sphere by design. In practice, we realize such APCD in an all-dielectric metasurface platform in the visible frequency range, manifesting transmissive polarization dichroism (PD) of nearly 100% in theory and greater than 90% experimentally. We exploit the perfect PD feature of this system to demonstrate arbitrary polarization, including linear, circular and elliptical polarization, directly from unpolarized light. This all-in-one metasurface polarizer serves as a monolithic arbitrary polarization generator, ultimately promising miniaturized optical devices for integrated nanophotonic systems with substantially reduced complexity.

## Results

**Fig. 1:**
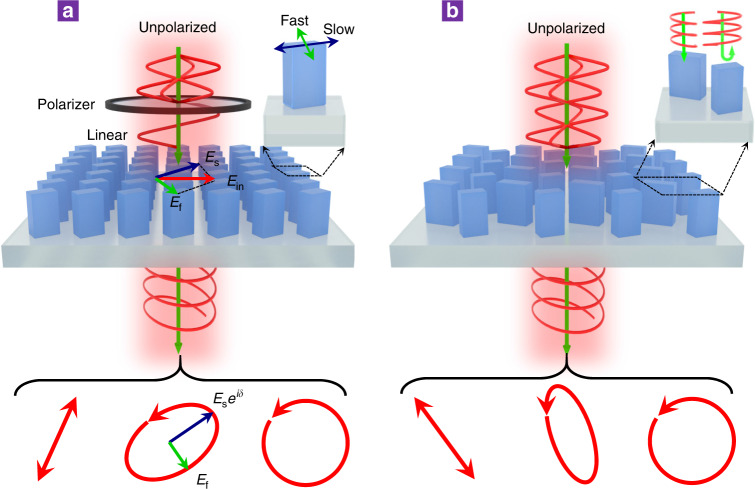
Comparison of the polarization controls based on the birefringence and dichroism metasurfaces. **a** Schematic of conventional polarization control based on a cascaded linear polarizer and birefringent single-meta-atom metasurface. The incident beam with well-defined polarization was converted to arbitrary polarization states by imparting different phase retardations on orthogonal linear polarizations along the fast and slow axes of the birefringent meta-atom. **b** Schematic of the proposed all-in-one polarizer that can function at an arbitrary position on the Poincaré sphere by design based on a dielectric metasurface composed of dimerized nanopillars, which can directly operate with unpolarized incident light and generate arbitrary polarization states, including linear, elliptical and circular polarizations, regardless of the incident polarization state.

We begin with conventional metasurfaces that manipulate polarization states through birefringent meta-atoms, which can impose distinct phase retardations along the fast and slow axes on two orthogonal linear polarizations, as shown in Fig. 1a. Such elementary meta-atoms can be described by the Jones matrix (in terms of electric field) in the linear polarization base as follows^41^:

1 $${\mathbf{J}}^{\mathbf{e}} = {\mathbf{R}}\left( \theta \right)\left( {\begin{array}{*{20}{c}} {e^{i\delta _f}} & 0 \\ 0 & {e^{i\delta _s}} \end{array}} \right){\mathbf{R}}\left( { - \theta } \right)$$

where δ_f_ and δ_s_ represent the phase retardations along the fast and slow axes of the birefringent meta-atoms, respectively, θ is the orientation angle of the fast axis, and $${\mathbf{R}}\left( \theta \right) = \left( {\begin{array}{*{20}{c}} {\cos \left( \theta \right)} & { - \sin \left( \theta \right)} \\ {\sin \left( \theta \right)} & {\cos \left( \theta \right)} \end{array}} \right)$$ denotes the rotation matrix. As seen from Eq. 1, the output polarization is heavily dependent upon the incident beam, which restricts its operation to well-defined incident polarizations that typically result from an additional linear polarizer. The proposed APCD metasurface can overcome the aforementioned intrinsic limitations and generate arbitrary polarization states–linear, circular, or elliptical–directly from unpolarized incident light. The metasurface consists of arrays of dimerized metamolecules containing two dielectric birefringent nanopillars, as schematically shown in Fig. 1b. Their far-field interference and collective contributions can be exquisitely tailored by the length, width, and orientation of the nanopillar pairs illuminated by different polarizations, leading to perfect transmissive dichroism for arbitrary orthogonal polarization pairs on demand. In this way, all-in-one metasurface polarizers that allow arbitrary polarization generation covering the full Poincaré sphere directly from unpolarized beams become feasible.

**Fig. 2:**
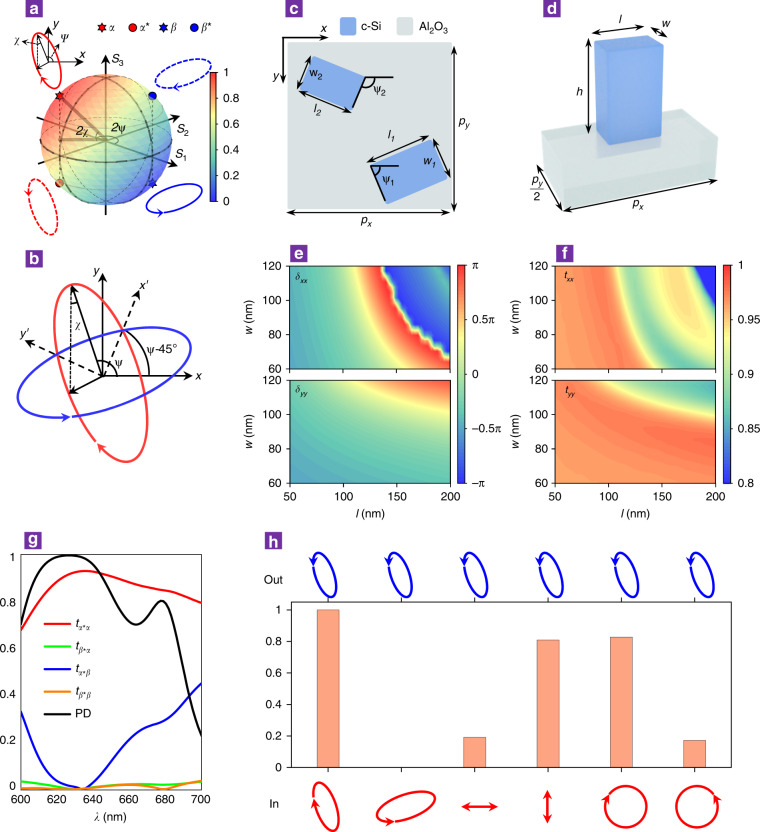
Metasurface design for APCD. **a** Poincaré sphere representation of an arbitrary orthogonal polarization pair (**α**, **β**) (solid red and blue ellipses) and its handedness-flipped pair (α*, β*) (dashed red and blue ellipses). **b** Defined local coordinate system *x’oy’* (dashed lines) that is rotated by *ψ*-45° with respect to the global *xoy* coordinate, in which the axes pass through cross points of the polarization ellipses of **α** and **β**. **c** Schematic of metamolecules on the metasurface consisting of asymmetrically dimerized nanopillars. The periods are *p*_*x*_ = *p*_y_ = 340 nm, and the orientation angles and positions of the two nanopillars are *ψ*_1_ = *ψ*-45° and *ψ*_2_ = *ψ* and (3*p*_*x*_/4, 3*p*_*y*_/4) and (*p*_*x*_/4, *p*_*y*_/4), respectively, where *ψ* is the main axis angle of the modulated polarization ellipse. **d** Single nanopillar unit cell for determining phase retardations along its length and width, as shown in **e**. The height of the nanopillar was fixed at 300 nm. **e** Phase retardations and **f** transmission coefficients upon varying the length *l* and width *w* of the nanopillar at a wavelength of 633 nm for the *x*-polarized (upper panels) and *y*-polarized (lower panels) incident light. **g** Polarization conversion coefficients and PD spectrum calculated with the optimized parameters (*l*_1_ = 130 nm, *w*_1_ = 70 nm; *l*_2_ = 150 nm, *w*_2_ = 85 nm) at a wavelength of 633 nm, which yields *t*_α*α_ = 0.93 and *t*_β*α_ = *t*_α*β_ = *t*_β*β_ ≈ 0. **h**, Transmitted polarization states (blue ellipses) when the metasurface was illuminated by a variety of different incident polarization states (red curves). The histogram indicates the transmittance, which varied with the incident polarization, while the shape of the blue ellipse was the same as that of the designed polarization state α*.

In general, an arbitrary polarization state **α** can be fully described by two parameters, namely, the main axis angle *ψ* and the ellipticity angle *χ* (Fig. 2a), which can be represented as a point with coordinates (2*ψ*, 2*χ*) on a Poincaré sphere (the red star in Fig. 2a). Its Jones vector can be explicitly written in terms of the parameters *ψ* and *χ* as follows:

2 $${\mathbf{\alpha }} = {\mathbf{R}}\left( \psi \right)\left( {\begin{array}{*{20}{c}} {\cos \left( \chi \right)} \\ { - i\sin \left( \chi \right)} \end{array}} \right){\mathrm{ = }}\frac{{\sqrt {\mathrm{2}} }}{{\mathrm{2}}}{\mathbf{R}}\left( {\psi - 45^\circ } \right)\left( {\begin{array}{*{20}{c}} {e^{i\chi }} \\ {e^{ - i\chi }} \end{array}} \right)$$

Its orthogonal polarization state **β** is located at the inversion symmetry point (the blue star in Fig. 2a) with coordinates (2(ψ−90°), − 2χ) and the following Jones vector:

3 $${\mathbf{\beta }} = {\mathbf{R}}\left( {\psi - 90^\circ } \right)\left( {\begin{array}{*{20}{c}} {\cos \left( \chi \right)} \\ {i\sin \left( \chi \right)} \end{array}} \right) = \frac{{\sqrt {\mathrm{2}} }}{{\mathrm{2}}}{\mathbf{R}}\left( {\psi - 45^\circ } \right)\left( {\begin{array}{*{20}{c}} {e^{i\chi }} \\ { - e^{ - i\chi }} \end{array}} \right)$$

As we can see, α and β can be written as the product of a rotation matrix with the same angle ψ − 45° and a Jones vector composed of only the parameter χ; therefore, we can define a local x’oy’ coordinate system that is rotated by ψ−45° with respect to the global xoy coordinate system (Fig. 2b) to simplify the deduction of the required Jones matrix for APCD (Supplementary Note 1). In addition, the handedness-flipped states α* and β* (where * denotes the complex conjugate operator) are located at mirror symmetry points with respect to the equatorial plane, as denoted by the red and blue dots, respectively, in Fig. 2a. To formulate the Jones matrix **J** for APCD that allows transmission and conversion of the polarization state **α** to its handedness-flipped polarization α* and completely blocks **β**, it is convenient to define another Jones matrix **J**^#^ connecting the input and output polarization states defined in the (**α**, **β**) base and (α*, β*) base, respectively. By applying a series of base transformations from a linear polarization base in the global xoy system to a linear polarization base in the local x’oy’ system and finally to an arbitrary polarization base, we can obtain **J**^#^ in terms of the parameters ψ and χ as follows (Supplementary Note 1):

4 $${\mathbf{J}}^{\mathbf{\# }} = \left( {\begin{array}{*{20}{c}} {t_{\alpha ^\ast \alpha }} & {t_{\beta ^\ast \alpha }} \\ {t_{\alpha ^\ast \beta }} & {t_{\beta ^\ast \beta }} \end{array}} \right) = \left( {\begin{array}{*{20}{c}} {e^{ - i\chi }} & {e^{ - i\chi }} \\ {e^{i\chi }} & { - e^{i\chi }} \end{array}} \right)^{ - 1}{\mathbf{R}}\left( {45^\circ - \psi } \right){\mathbf{JR}}\left( {\psi - 45^\circ } \right)\left( {\begin{array}{*{20}{c}} {e^{i\chi }} & {e^{i\chi }} \\ {e^{ - i\chi }} & { - e^{ - i\chi }} \end{array}} \right)$$

where the matrix elements t_ji_ (i = α, β; j = α*, β*) represent the conversion coefficients from polarization state **i** to state **j**. Perfect APCD requires t_α*α_ = 1, and t_β*α_ = t_α*β_ = t_β*β_ = 0. Upon substituting those conditions into Eq. 4, we can obtain the Jones matrix **J** in the xoy coordinate as follows (Supplementary Note 1):

5 $${\mathbf{J}} = \frac{1}{2}{\mathbf{R}}\left( {\psi - 45^\circ } \right)\left( {\begin{array}{*{20}{c}} {e^{ - 2i\chi }} & 1 \\ 1 & {e^{2i\chi }} \end{array}} \right){\mathbf{R}}\left( {45^\circ - \psi } \right)$$

To achieve its physical implementation with explicit birefringent meta-atoms as described by Eq. 1, Eq. 5 can be decomposed as the superposition of two linear birefringent waveplates (Supplementary Note 1), as follows:

6 $${\mathbf{J}} = \frac{1}{2}{\mathbf{R}}\left( {\psi - 45^\circ } \right)\left( {\begin{array}{*{20}{c}} {e^{ - 2i\chi }} & 0 \\ 0 & {e^{2i\chi }} \end{array}} \right){\mathbf{R}}\left( {45^\circ - \psi } \right) + \frac{1}{2}{\mathbf{R}}\left( \psi \right)\left( {\begin{array}{*{20}{c}} 1 & 0 \\ 0 & { - 1} \end{array}} \right){\mathbf{R}}\left( { - \psi } \right)$$

The first term in Eq. 6 can be physically realized by a birefringent meta-atom that has an orientation angle of ψ−45° and imposes phase shifts of −2χ and 2χ along its fast and slow axes, respectively. The second term has phase retardations of 0 and π along the fast and slow axes, respectively, and an orientation angle of ψ.

In the practical metasurface design, we use high-index all-dielectric nanopillars composed of crystalline silicon (c-Si) on top of an Al_2_O_3_ substrate as our building blocks (Fig. 2c). Once the polarization parameters (*ψ*, *χ*) for dichroism are determined, the two nanopillars with properly tailored lengths *l* and widths *w* will be selected for meta-molecule designs. To generate the lookup table for birefringent meta-atoms with optimal geometries to satisfy Eq. 6, we first examine the amplitude and phase of light transmitted from a metasurface composed of periodic arrays of single nanopillars, as shown in Fig. 2d. The amplitude and phase responses calculated from periodic arrays are used to approximate those meta-atoms in Eq. 6. The imposed phase retardations (*δ*_*xx*_ and *δ*_*yy*_) and transmission coefficients (*t*_*xx*_ and *t*_*yy*_) for the *x*- and *y*-polarized beams are determined through numerical simulations by varying the geometric dimensions of the nanopillars, as depicted in Fig. 2e, f. Distinct phase retardations along the fast and slow axes of the nanopillars can be flexibly configured by properly designing their lengths and widths while maintaining transmission coefficients greater than 90%.

Without loss of generality, we first designed a metasurface that allows preferential transmission of the polarization state **α** (*ψ* = 112.5°, *χ* = 22.5°) and completely rejects its orthogonal state **β** (*ψ−*90° = 22.5°*, −χ* = −22.5°). Based on the above analysis, optimal geometric parameters of *l*_*1*_ = 130 nm, *w*_*1*_ = 70 nm, *l*_*2*_ = 150 nm, *w*_*2*_ = 85 nm and *h* = 300 nm were chosen for the elliptical polarization conversion dichroism. The arrangement of the meta-atoms is shown in Fig. 2c, where the centre positions of the two nanopillars were optimized to (3*p*_*x*_/4, *3p*_*y*_/4) and (*p*_*x*_/4, *p*_*y*_/4) (Supplementary Note 1 and Fig. S2). To characterize the dichroism performance, we defined the transmissive PD as the relative difference in transmittances of polarization states **α** and **β**,

7 $${\mathrm{PD}} = \frac{{T_\alpha - T_\beta }}{{T_\alpha + T_\beta }}$$

The calculated polarization conversion coefficients (defined as matrix elements of **J**^#^ in Eq. 4) as well as the PD spectrum are plotted in Fig. 2g as a function of wavelength. Here, the polarization conversion coefficient of t_α*α_ is 0.93, and the other three coefficients are completely suppressed at a wavelength of 633 nm, resulting in a peak value of PD close to unity (black curve in Fig. 2g), indicating perfect polarization conversion dichroism for the orthogonal elliptical polarization pair (**α**, **β**). This metasurface serves as a perfect arbitrary polarizer that allows a specific elliptical polarization to pass through, regardless of the polarization state of the incident light. As shown in Fig. 2h, for incidence light with various input polarizations (denoted by red ellipses, lines and circles), the polarization of the transmitted light (blue ellipses) maintains the same state α* (ψ = 112.5°, −χ = −22.5°), while the transmittance is determined by the projection of the incident polarization states on α in the polarization base vector (**α**, **β**), as shown in the histograms.

**Fig. 3:**
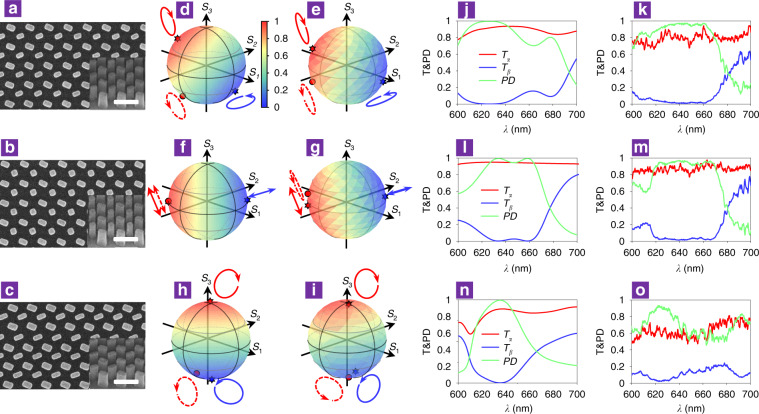
Experimental demonstration of the elliptical, linear and circular polarization conversion dichroism. **a**–**c** SEM images of the dichroism metasurfaces for different ellipticities, including (**a**) elliptical, (**b**) linear and (**c**) circular polarization states; the scale bar is 400 nm. The sizes (*l*_*1*_, *w*_*1*_, *l*_*2*_, *w*_*2*_) of the two nanopillars are (130 nm, 70 nm, 150 nm, 85 nm), (95 nm, 95 nm, 145 nm, 80 nm) and (160 nm, 75 nm, 165 nm, 100 nm) for **a**, **b** and **c**, respectively. **d–i** Simulated and measured transmittances of all possible polarizations covering the full Poincaré sphere through the **d, e** elliptical, **f, g** linear and **h, i** circular dichroism metasurfaces. The red and blue stars represent the polarization states with maximum and minimum transmittance depicted as red and blue ellipses, respectively. The dots represent the transmitted polarization states denoted as dashed ellipses. **j–o** Simulated and measured transmission spectra and dichroism spectra for **j, k** elliptical, **l, m** linear and **n, o** circular dichroism metasurfaces.

The freedom provided by the proposed metasurface allows full Poincaré sphere polarization control. To demonstrate the viability and versatility, we experimentally fabricated three dichroic metasurfaces to achieve three kinds of polarization: elliptical (*ψ* = 112.5°, *χ* = 22.5°), linear (*ψ* = 112.5°, *χ* = 0), and circular (*ψ* = 112.5°, *χ* = 45°). The corresponding scanning electron microscopy (SEM) images are shown in Fig. 3a–c. Figure 3d, e shows the simulated and experimentally measured transmittance of the elliptical polarization conversion dichroism metasurface illuminated by all possible polarization states marked in the Poincaré sphere. The incident polarization states with maximum and minimum transmittance values are plotted as the red and blue stars and the red and blue ellipses, respectively, which are consistent with the corresponding elliptical polarization states **α** and **β** that are allowed and blocked by the metasurface design. The dashed red ellipse indicates the polarization state α* of the output beam with flipped handedness and almost identical ellipticity as the incident polarization **α**, which unambiguously verifies the elliptical polarization conversion dichroism. We note that the polarization states in the experiment deviated slightly from those of the simulation, which can be attributed to fabrication imperfection. The PD spectrum shows a peak close to unity in the simulation (Fig. 3j) and greater than 95% in the experiment (Fig. 3k). Benefitting from such high dichroism performance, it can generate an elliptical polarization state α* independent of the incident polarization, which is presented in Supplementary Note 2 and Fig. S3.

Similarly, the transmittance of the linear and circular polarization conversion dichroism metasurfaces under illumination by all possible polarization states covering the Poincaré sphere is shown in Fig. 3f–g and Fig. 3h–i, respectively. The incident polarization states with maximum and minimum transmittances are distributed on the opposite site of the Poincaré sphere, representing two pairs of orthogonal polarizations, namely, linear and circular polarization states, respectively. Likewise, the PDs are close to unity in the simulation and greater than 95% for the linear polarization (Fig. 3l, m) and 90% for the circular polarization (Fig. 3n–o) in the experiment. Notably, the APCD metasurfaces also support AT phenomena beyond linear and circular polarization^16,47^, as demonstrated in Supplementary Note 3 and Fig. S4. The allowed and prevented polarization states of the APCD metasurface can be switched by simply swapping the length and width parameters of either of the two nanopillars, as shown in Supplementary Note 4 and Fig. S5. Beyond the three abovementioned representative polarization conversion dichroisms, APCD designs covering the full Poincaré sphere can be implemented by means of the procedure introduced in Supplementary Note 5 and Figs. S6, S7. First, we can achieve polarization conversion dichroism along the latitude lines of the Poincaré sphere with different ellipticities χ by sweeping nanopillar dimensions. Then, we can simply rotate the entire metasurface to realize another polarization conversion dichroism along the longitude lines on the Poincaré sphere, which represent polarization states with an identical ellipticity χ but different *ψ*. By combining the above two steps, APCD covering the full Poincaré sphere can be obtained.

**Fig. 4:**
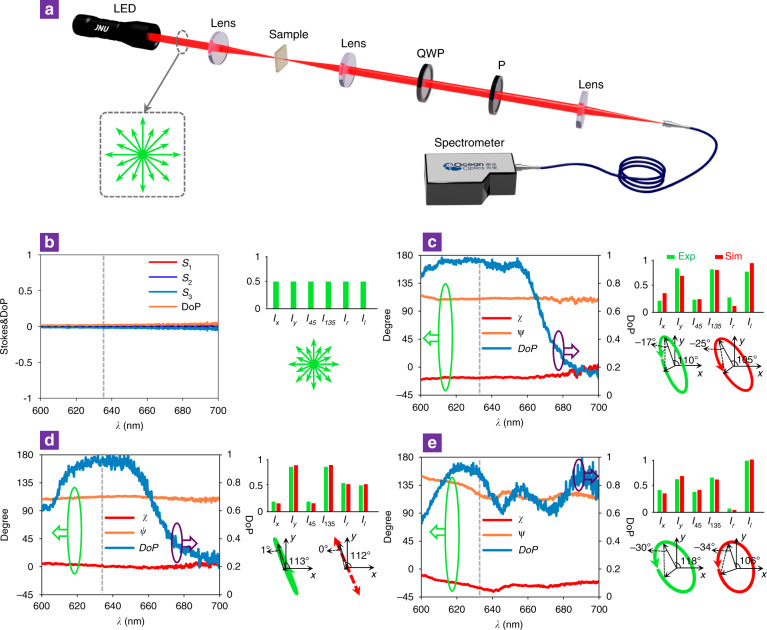
Demonstration of all-in-one APCD metasurfaces acting on unpolarized incident light. **a** Experimental setup for the *DoP* measurement of transmitted light from APCD metasurfaces illuminated by an unpolarized LED source. **b** Measured Stokes parameters and the *DoP* of the incident beam directly from the LED source. The histogram shows the measured intensities of different electric components at a wavelength of 633 nm. The unpolarized light beam is represented by the green arrows. **c–e** Measured main axis angle *ψ*, ellipticity angle *χ* and *DoPs* of the transmitted beams through the designed **c** elliptical, **d** linear and **e** circular dichroism metasurfaces. The histograms show the measured (green) and simulated (red) intensities of different electric components at a wavelength of 633 nm. The green polarization ellipses represent polarization states derived from measured Stokes parameters at 633 nm, which are consistent with the simulated states (red arrows).

As a proof of principle, we exploit APCD to demonstrate direct polarization generation from unpolarized light. Figure 4a shows the experimental configuration, where an LED is employed as the unpolarized light source. The polarization state of the transmitted beam can be obtained by measuring the Stokes parameters. Figure 4b shows the measured Stokes parameters and the degree of polarization (*DoP*, defined as $\sqrt {{\mathrm{S}}_1^2 + {\mathrm{S}}_2^2 + {\mathrm{S}}_3^2}$) directly from the LED beam source. Indeed, the polarization state directly from the LED source is undefined. After passing through the monolithic APCD metasurfaces, the measured *DoP*s are close to unity, indicating a well-defined polarization state centred at a wavelength of 633 nm (Fig. 4c–e). The output polarizations can be obtained by analysing the Stokes parameters derived from the measured intensities of four linear polarizations polarized along *x*, *y*, 45°, and 135° and two circular polarizations in the transmitted beams, as shown in the histograms in Fig. 4c–e, which are depicted by the polarization ellipses determined by the main axis angle *ψ* and the ellipticity angle *χ*. The measured output polarization states (green) show reasonable congruence with the simulated state (red). Although the main axis angle *ψ* and the ellipticity angle *χ* of the output polarization states at other wavelengths exhibit deviate slightly from the ideal case at 633 nm, the *DoPs* are measured to maintain a value above 0.9 over a bandwidth for linear, elliptical and circular dichroism metasurfaces, promising high-performance polarizers.

## Discussion

In this paper, we have proposed a general design for all-dielectric metasurfaces that can realize perfect APCD and demonstrated their applicability for all-in-one polarizers that function at an arbitrary position on the Poincaré sphere by design. The proposed APCD metasurfaces are composed of asymmetrically dimerized birefringent dielectric nanopillars with certain intersection angles, enabling us to exquisitely configure their far-field interference for perfect dichroism close to 100% in the simulation and greater than 90% in experiments for arbitrary polarizations, including linear, circular or elliptical polarizations. The advanced features and design flexibility of the proposed metasurface offer the potential for the development of all-in-one metasurface polarizers directly operating with unpolarized beams for full Poincaré sphere polarization manipulations, which can dramatically encourage work on state-of-the-art planar optics and extensively promote integrated devices based on meta-optics.

## Materials and methods

### Theoretical design of the APCD metasurface

Numerical simulations were carried out by using the finite element method (FEM). First, polarization conversion coefficients and phase retardations under illumination by linearly polarized beams were calculated, with periodic boundary conditions applied in both the *x* and *y* directions. Second, the Jones matrix was obtained by analysing the results from the FEM simulation. The transmittance under all polarization states covering the full Poincaré sphere was obtained by multiplying the polarization state of the incident light by the Jones matrix, where incident beams with different polarizations were superposed by the two orthogonal linear-polarized base vectors.

### Experimental fabrication of an APCD metasurface

The metasurface was fabricated on commercially available 300-nm thick c-Si (100) epitaxially grown on a sapphire substrate (from UniversityWafer, Inc.). The structure was patterned on a positive resist (PMMA A2) using an E-beam writer (Raith E-line, 30 kV). After developing the resist, we transferred the pattern from the resist onto a chromium film with a thickness of 25 nm. Then, the silicon layer was etched by inductively coupled plasma etching (PlasmaPro System 100ICP180) using chromium as a hard mask. The remaining Cr was removed with a chromium etchant.

### Characterization of the APCD metasurface

A supercontinuum laser (Fianium-WL-SC480) was employed as the broadband light source for measurement of the transmission spectra under a specific polarization incidence, while an LED was used for unpolarized incident light. Incident light with a specific polarization located at an arbitrary position on the Poincaré sphere was generated by cascading a broadband linear polarizer and quarter waveplate from the supercontinuum laser. Then, the incident light was focused on the sample by a lens with a focal length of 10 cm. The transmittance and Stokes parameters were determined by means of an Ocean spectrometer (USB4000).

## Supplementary information

Supplemental imformation

## Supplementary information

**Supplementary information** The online version contains supplementary material available at 10.1038/s41377-021-00468-y.
