# Supplementary material for: Arbitrary polarization conversion dichroism metasurfaces for all-in-one full Poincaré sphere polarizers
Source: Light Sci Appl. 2021 Jan 27;10:24. doi: 10.1038/s41377-021-00468-y (PMC7841175; doi:10.1038/s41377-021-00468-y)
Supplement: Supplementary file 1 — Supplemental imformation [file 41377_2021_468_MOESM1_ESM.docx]

Supplementary Information for

Arbitrary Polarization Conversion Dichroism Metasurfaces for All-in-One Full Poincaré Sphere Polarizers

Shuai Wang^1, †^, Zi-Lan Deng^1, †^, *, Yujie Wang^2, †^, Qingbin Zhou^1^, Xiaolei Wang^3^, Yaoyu Cao^1^, Bai-Ou Guan^1^, Shumin Xiao^2^, *, Xiangping Li^1^, *

^1^Guangdong Provincial Key Laboratory of Optical Fiber Sensing and Communications, Institute of Photonics Technology, Jinan University, Guangzhou 510632, China.

^2^Ministry of Industry and Information Technology Key Lab of Micro-Nano Optoelectronic Information System, Harbin Institute of Technology, Shenzhen 518055, China

^3^Institute of Modern Optics, Nankai University, Tianjin 300350, China

^*^E-mail: [zilandeng@jnu.edu.cn](mailto:zilandeng@jnu.edu.cn), [shumin.xiao@hit.edu.cn](mailto:shumin.xiao@hit.edu.cn), [xiangpingli@jnu.edu.cn](mailto:xiangpingli@jnu.edu.cn).

^†^These authors contributed equally to this work.

**Note 1: Jones matrix analysis of arbitrary polarization conversion dichroism**

An arbitrary elliptical polarization state **α** can be fully described by its main axis angle *ψ* and ellipticity angle *χ*, which can be represented by a point (2*ψ*, 2*χ*) on a Poincaré sphere (red star in Figure 2a in the main text). In the global linear polarization base defined in the *xoy* coordinate system, the Jones vector of **α** can be explicitly written as follows in terms of (*ψ*, *χ*):

$\mathbf{\alpha}=\mathbf{R}\left(\psi\right)\left(\begin{matrix} \cos\left(\chi\right) \\ -i\sin\left(\chi\right) \end{matrix}\right)\text{=}\frac{\sqrt{2}}{2}\mathbf{R}\left(\psi-45^{\circ}\right)\left(\begin{matrix} e^{i\chi} \\ e^{-i\chi} \end{matrix}\right)$ (S1)

where, $\mathbf{R}\left(\psi\right)=\left(\begin{matrix} \cos\left(\psi\right) & -\sin\left(\psi\right) \\ \sin\left(\psi\right) & \cos\left(\psi\right) \end{matrix}\right)$ denotes the rotation matrix.

Its orthogonal polarization state **β** is located at the point (2(*ψ-*90°), -2*χ*) of the Poincaré sphere, with Jones vector as follows:

$\mathbf{\beta}=\mathbf{R}\left(\psi-90^{\circ}\right)\left(\begin{matrix} \cos\left(\chi\right) \\ i\sin\left(\chi\right) \end{matrix}\right)=\frac{\sqrt{2}}{2}\mathbf{R}\left(\psi-45^{\circ}\right)\left(\begin{matrix} e^{i\chi} \\ -e^{-i\chi} \end{matrix}\right)$ (S2)

The Jones matrix **J** relates the complex amplitudes of the incident field **i** ***=*** (*i_x_*, *i_y_*)^T^ to the complex amplitudes of the transmitted field **t** = (*t_x_*, *t_y_*)^T^ in the *xoy* coordinate as follows:

$\left(\begin{matrix} t_{x} \\ t_{y} \end{matrix}\right)\text{=}\mathbf{J}\left(\begin{matrix} i_{x} \\ i_{y} \end{matrix}\right)$ (S3)

For studying the polarization conversions between the incident and transmitted beams through the APCD metasurface, we define Jones vectors $\hat{\mathbf{i}}$ and $\hat{\mathbf{t}}$ in the polarization pair bases of (**α**, **β**) and (**α***, **β***), respectively. They are related with Jones vectors defined in the global linear base as follows^1^,

$\mathbf{i}=\mathbf{\Lambda}_{\mathbf{i}}\hat{\mathbf{i}}=\left(\begin{matrix} \mathbf{\alpha} & \mathbf{\beta} \end{matrix}\right)\hat{\mathbf{i}}$ (S4)

$\mathbf{t}=\mathbf{\Lambda}_{\mathbf{t}}\hat{\mathbf{t}}=\left(\begin{matrix} \mathbf{\alpha}^{\mathbf{*}} & \mathbf{\beta}^{*} \end{matrix}\right)\hat{\mathbf{t}}$ (S5)

where, the $\mathbf{\Lambda}_{\mathbf{k}}$ (k = i, t) is the base transformation matrix. Substituting Equation S4 and S5 into Equation S3, we obtain,

$\mathbf{t}=\mathbf{Ji}\to\mathbf{\Lambda}_{\mathbf{t}}\hat{\mathbf{t}}=\mathbf{J\Lambda}_{\mathbf{i}}\hat{\mathbf{i}}\to\hat{\mathbf{t}}=\mathbf{\Lambda}_{\mathbf{t}}^{\mathbf{-1}}\mathbf{J\Lambda}_{\mathbf{i}}\hat{\mathbf{i}}$ (S6)

therefore, we can define another Jones matrix **J^#^**:

$\mathbf{J}^{\mathbf{\#}}=\mathbf{\Lambda}_{\mathbf{t}}^{\mathbf{-1}}\mathbf{J\Lambda}_{\mathbf{i}}$ (S7)

to connect the complexed amplitudes of the incident and transmitted light in the polarization pair bases of (**α**, **β**) and (**α***, **β***), respectively, as follows,

$\left(\begin{matrix} t_{\alpha^{*}} \\ t_{\beta^{*}} \end{matrix}\right)=\mathbf{J}^{\mathbf{\#}}\left(\begin{matrix} i_{\alpha} \\ i_{\beta} \end{matrix}\right)$ (S8)

To deduce the Jones matrix for arbitrary polarization conversion dichroism (APCD), it is advantageous to define a local coordinate system *x'oy'* such that the cross points of the two orthogonal polarization ellipses (**α** and **β**) just lie on its axes o*x'* and *oy'* respectively. The *x'oy'* coordinate is clockwise rotating by *ψ*-45° with respect to the original *xoy* system, as shown in Figure 2b of the main text. In the local *x'oy'* coordinate, the parameter *ψ* is eliminated in their Jones vectors as follows:

$\mathbf{\alpha'}=\mathbf{R}\left(-\left(\psi-45^{o}\right)\right)\mathbf{\alpha}=\frac{\sqrt{2}}{2}\left(\begin{matrix} e^{i\chi} \\ e^{-i\chi} \end{matrix}\right)$ (S9)

$\mathbf{\beta'}=\mathbf{R}\left(-\left(\psi-45^{o}\right)\right)\mathbf{\beta}=\frac{\sqrt{2}}{2}\left(\begin{matrix} e^{i\chi} \\ -e^{-i\chi} \end{matrix}\right)$ (S10)

The polarization states with flipped handedness to **α'** and **β'** can be described in the *x'oy'* coordinate as follows:

$\mathbf{\alpha'}^{\mathbf{*}}=\frac{\sqrt{2}}{2}\left(\begin{matrix} e^{-i\chi} \\ e^{i\chi} \end{matrix}\right)$ (S11)

$\mathbf{\beta'}^{\mathbf{*}}=\frac{\sqrt{2}}{2}\left(\begin{matrix} e^{-i\chi} \\ -e^{i\chi} \end{matrix}\right)$ (S12)

In the *x'oy'* coordinate, the Jones matrix **J'** relates the incident field **i'** = (*i_x_*', *i_y_*')^T^ to the transmitted field **t' =** (*t_x_'*, *t_y_'*)^T^ following the same way:

$\left(\begin{matrix} t_{x}^{'} \\ t_{y}^{'} \end{matrix}\right)\text{=}\mathbf{J'}\left(\begin{matrix} i_{x}^{'} \\ i_{y}^{'} \end{matrix}\right)$ (S13)

According to the base vector transformation between the global linear polarization base (*xoy*) and the local linear polarization base (*x'oy'*), the relationship between **J** and **J'** is,

$\mathbf{J}=\mathbf{R}\left(\psi-45^{\circ}\right)\mathbf{J'R}\left(45^{\circ}-\psi\right)$ (S14)

As we consider planar chiral structures with mirror symmetry in the propagation direction, the Jones matrix **J'** is a symmetric matrix as follows^2^:

$\mathbf{J'}=\left(\begin{matrix} a & b \\ b & c \end{matrix}\right)$ (S15)

According to Equation S7, transforming the Jones matrix **J'** in local linear polarization base to the Jones matrix **J^#^** in the arbitrary polarization base yields,

$\begin{matrix} \mathbf{J}^{\mathbf{\#}}=\left(\begin{matrix} A & B \\ C & D \end{matrix}\right)=\left(\begin{matrix} \mathbf{\alpha'}^{\mathbf{*}} & \mathbf{\beta'}^{\mathbf{*}} \end{matrix}\right)^{-1}\mathbf{J'}\left(\begin{matrix} \mathbf{\alpha'} & \mathbf{\beta} \end{matrix}\mathbf{'}\right) \\ =\left(\begin{matrix} e^{-i\chi} & e^{-i\chi} \\ e^{i\chi} & -e^{i\chi} \end{matrix}\right)^{-1}\mathbf{J'}\left(\begin{matrix} e^{i\chi} & e^{i\chi} \\ e^{-i\chi} & -e^{-i\chi} \end{matrix}\right) \\ =\frac{1}{2}\left(\begin{matrix} ae^{2i\chi}+2b+ce^{-2i\chi} & ae^{2i\chi}-ce^{-2i\chi} \\ ae^{2i\chi}-ce^{-2i\chi} & ae^{2i\chi}-2b+ce^{-2i\chi} \end{matrix}\right) \end{matrix}$ (S16)

Combining Equation S14 and Equation S16, we obtain Equation 4 in the main text.

Perfect APCD requires that,

$A=\frac{1}{2}\left(ae^{2i\chi}+2b+ce^{-2i\chi}\right)=1$ (S17)

$B=C=\frac{1}{2}\left(ae^{2i\chi}-ce^{-2i\chi}\right)=0$ (S18)

$D=\frac{1}{2}\left(ae^{2i\chi}-2b+ce^{-2i\chi}\right)=0$ (S19)

which yield,

$\text{a=}\frac{1}{2}e^{-2i\chi}$, $\text{b=}\frac{1}{2}$, $\text{c=}\frac{1}{2}e^{2i\chi}$ (S20)

Substituting Equation S20 to Equation S15, we obtain the final form of Jones matrix **J'** in the local *x'oy'* coordinate system as:

$\mathbf{J'}=\frac{1}{2}\left(\begin{matrix} e^{-2i\chi} & 1 \\ 1 & e^{2i\chi} \end{matrix}\right)$ (S21)

Substituting Equation S21 back into Equation S14, we obtain the Jones matrix **J** in the global *xoy* coordinate system as:

$\mathbf{J}=\frac{1}{2}\mathbf{R}\left(\psi-45^{\circ}\right)\left(\begin{matrix} e^{-2i\chi} & 1 \\ 1 & e^{2i\chi} \end{matrix}\right)\mathbf{R}\left(45^{\circ}-\psi\right)$ (S22)

which is the same as Equation 5 in the main text. To its physical realization of the Jones matrix by explicit meta-atom designs, we can split Equation. S22 into two terms as follows,

$$\mathbf{J}=\frac{1}{2}\mathbf{R}\left(\psi-45^{\circ}\right)\left(\begin{matrix} e^{-2i\chi} & 0 \\ 0 & e^{2i\chi} \end{matrix}\right)\mathbf{R}\left(45^{\circ}-\psi\right)+\frac{1}{2}\mathbf{R}\left(\psi\right)\mathbf{R}\left(-45^{\circ}\right)\left(\begin{matrix} 0 & 1 \\ 1 & 0 \end{matrix}\right)\mathbf{R}\left(45^{\circ}\right)\mathbf{R}\left(-\psi\right)$$

$\text{=}\frac{1}{2}\mathbf{R}\left(\psi-45^{\circ}\right)\left(\begin{matrix} e^{-2i\chi} & 0 \\ 0 & e^{2i\chi} \end{matrix}\right)\mathbf{R}\left(45^{\circ}-\psi\right)+\frac{1}{2}\mathbf{R}\left(\psi\right)\left(\begin{matrix} 1 & 0 \\ 0 & -1 \end{matrix}\right)\mathbf{R}\left(-\psi\right)$ (S23)

which is same as Equation 6 in the main text. Thus, the Jones matrix can be realized by a pair of birefringent meta-atoms. The first one has phase retardations -2*χ* and 2*χ* along the fast and slow axes, respectively, and an orientation angle *ψ-*45^o^; while the second one has phase retardations 0 and π along the fast and slow axes, respectively, and an orientation angle of *ψ*. Once a pair of orthogonal polarization states is given, we can first select dielectric nanopillars with targeted phase retardations from the look-up table depicted in Figure 2(e), and then combine the two nanopillars as a meta-molecule for further optimization. Finally, we obtained the dimerized metasurface design to realize perfect dichroism for a given polarization pair.


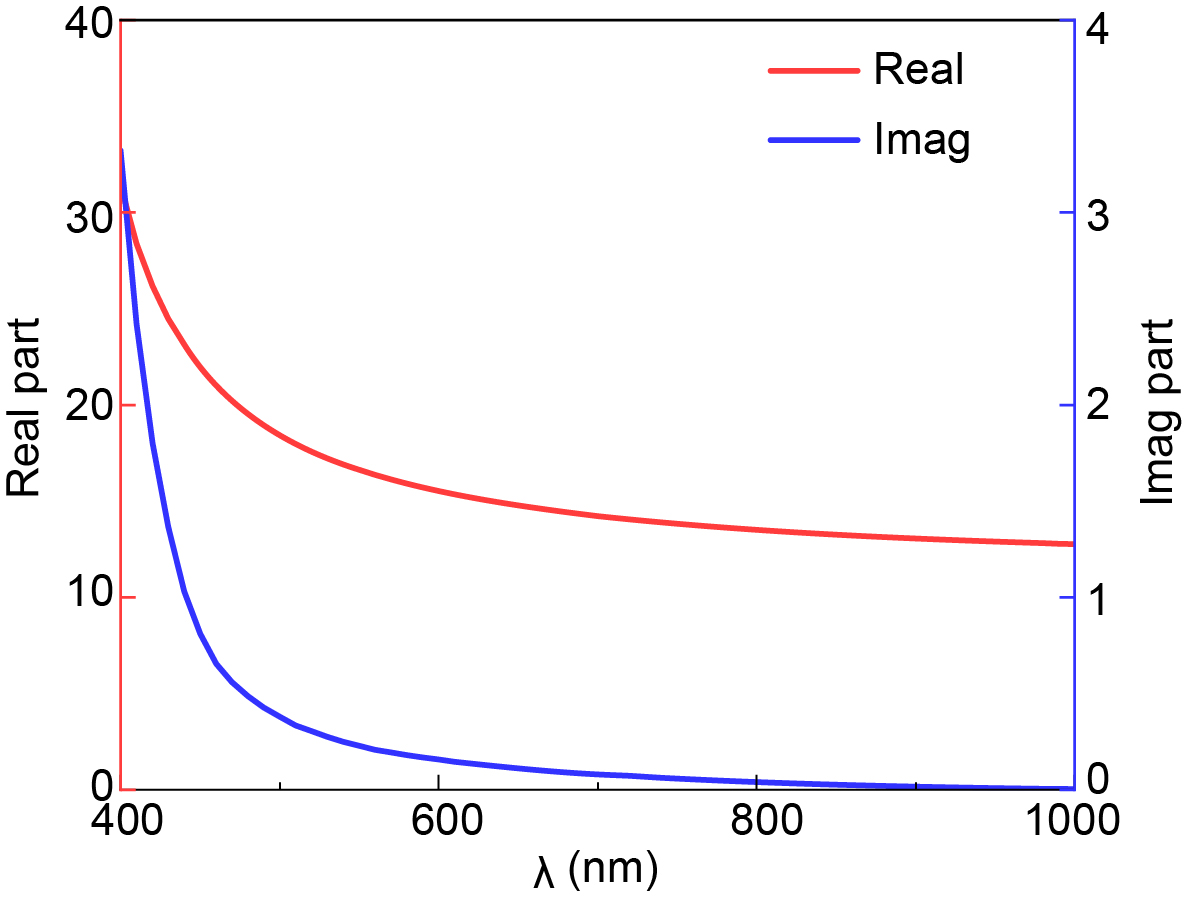


**Figure S1** the permittivity curves of the c-Si measured by the ellipsometer

The dielectric material of c-Si with permittivities shown in Figure. S1 is used to design the dichroism metasurface with relatively small loss in the visible range. Once the two nanopillars with optimal geometries are chosen, we can realize the dimerized metasurface to fulfill the designed Jones matrix. To avoid near-field coupling between the two nanopillars, the positions of the two nanopillars are optimized so that each nanopillar works as independent meta-atoms. Here we take the elliptical dichroism metasurface (the one in Figure 2 of the main text) as an example, where the positions of the two nanopillars are (3*p_x_*/4, *3p_y_*/4) and (*p_x_*/4, *p_y_*/4), respectively. Figure S2b and c shows the electric field *E_x_* distribution under illumination by the incident polarization beams of **α** and **β** in the cutting surface shown as the red plane in Figure S2a, where the electric fields are well confined inside and around the high index dielectric nanopillars with negligible near-field couplings. Therefore, the two nanopillars work as two independent birefringent waveplates allowing far-field interferences. Further, we examine the performance of the metasurface at varied distances between centers of the two nanopillars. The transmittances and PDs under illumination by incident polarizations of **α** and **β** for reducing the distance from 240 nm to 80 nm are shown in Figure S2d-e. It clearly shows that the transmittances have little variations for the distance lager than 160 nm, where the PDs are always close to 100% at the designed wavelength of 633 nm. Thus, the distance is fixed at 240 nm throughout the manuscript in both simulations and experiments. The Jones matrix for APCD realized by a pair of birefringent meta-atoms has been demonstrated.


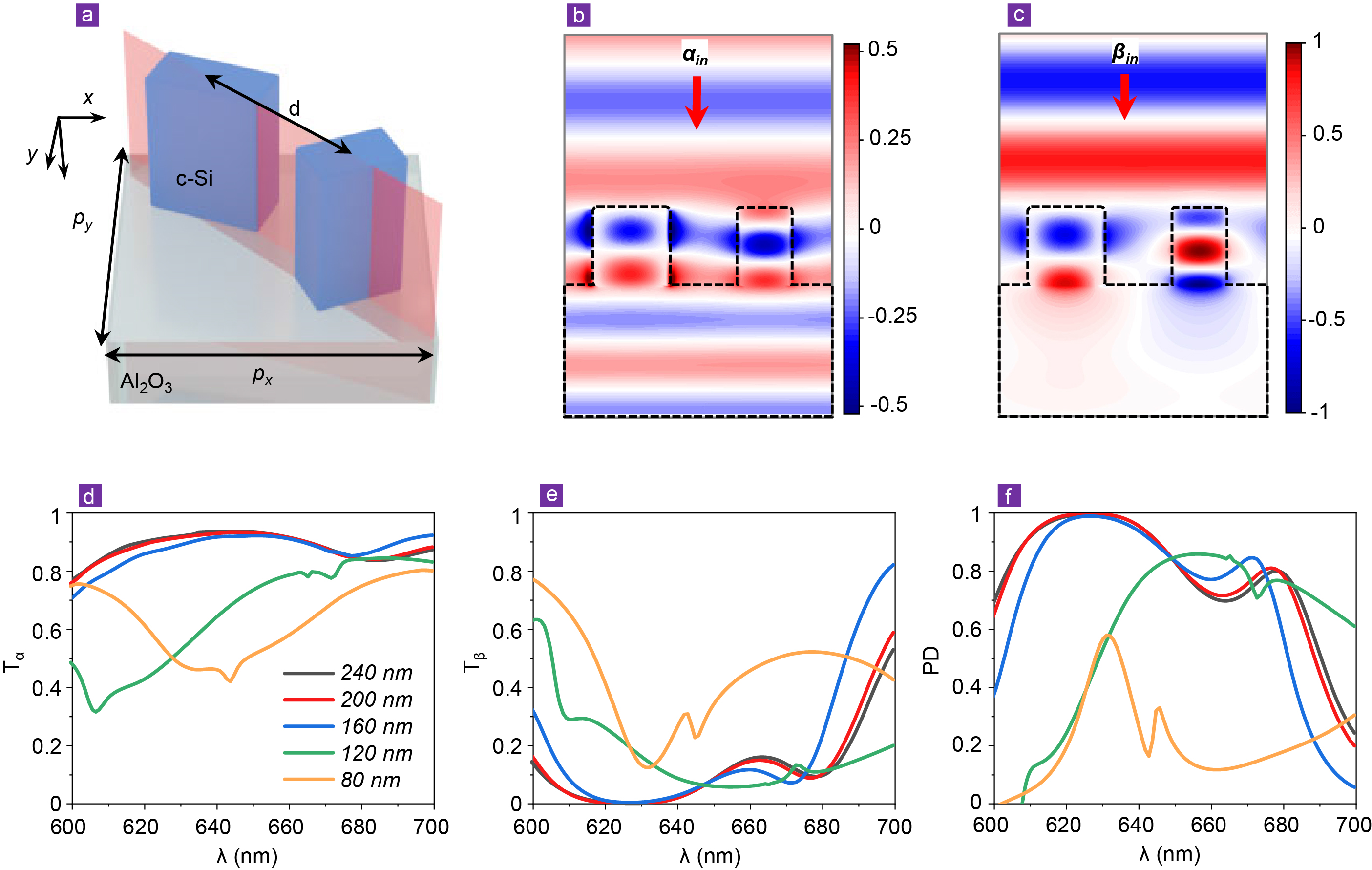


**Figure S2**. Meta-molecule design by two birefringent nanopillars to realize the Jones matrix for APCD. **a,** Illustration of the arrangement of the two birefringent nanopillars in the meta-molecule. **b, c,** The electric field *E_x_* under illumination by polarization states of ***α*** and ***β*** at the designed wavelength of 633 nm on the cutting surface (red plane in Figure S2a), respectively. The red arrows represent the incident directions, the dash frames represent the position of the nanostructure, where the distance between centers of the two nanopillars is d = $\sqrt{2}p_{x}\approx$ 240 nm. **d**-**f,** The transmittances of incident beams of **α** and **β** and PD spectra varying as a function of distance between the centers of the two meta-atoms.

**Note 2: Output transmittances and polarization states under arbitrary polarization incidences covering the full Poincaré sphere.**

As the transmittance through APCD metasurface is determined by the projection of the incident polarization states on the polarization state **α** in the polarization base vectors (**α***,* **β**). To examine the transmittance and output polarizations through the designed metasurface, we can define a new set of parameters as [*S_1_'* *S_2_' S_3_'*] = [*S_1_* *S_2_ S_3_*]∙T , where [*S_1_* *S_2_ S_3_*] are Stokes parameters of the transmitted beam through the metasurfaces. [*S_1_'* *S_2_' S_3_'*] are the overall transmittances of transmitted polarization state [*S_1_* *S_2_ S_3_*]. The [*S_1_'* *S_2_' S_3_'*] forms a spindle-like surface (Figure S3c-e). The long axis direction of the spindle indicates the polarization state with the largest transmittance, indicating that the transmitted polarization states converge into the designed output polarization under arbitrary polarization incidences. At the idea situation, the spindle should be close to a volume-less line (dashed black line). In practice, the smaller the spindle volume, the better the polarizer performance.

Figures S3d,e show the simulated and experimental results of the output polarization states of the practical realized elliptical dichroism metasurfaces under a series of linearly polarized incident beams with different orientation angles as shown in the legend. It can be seen that the output polarization states indeed converge at the designed one. The slight variation around the desired output polarization is due to the imperfect dichroism at the presence of the substrate.


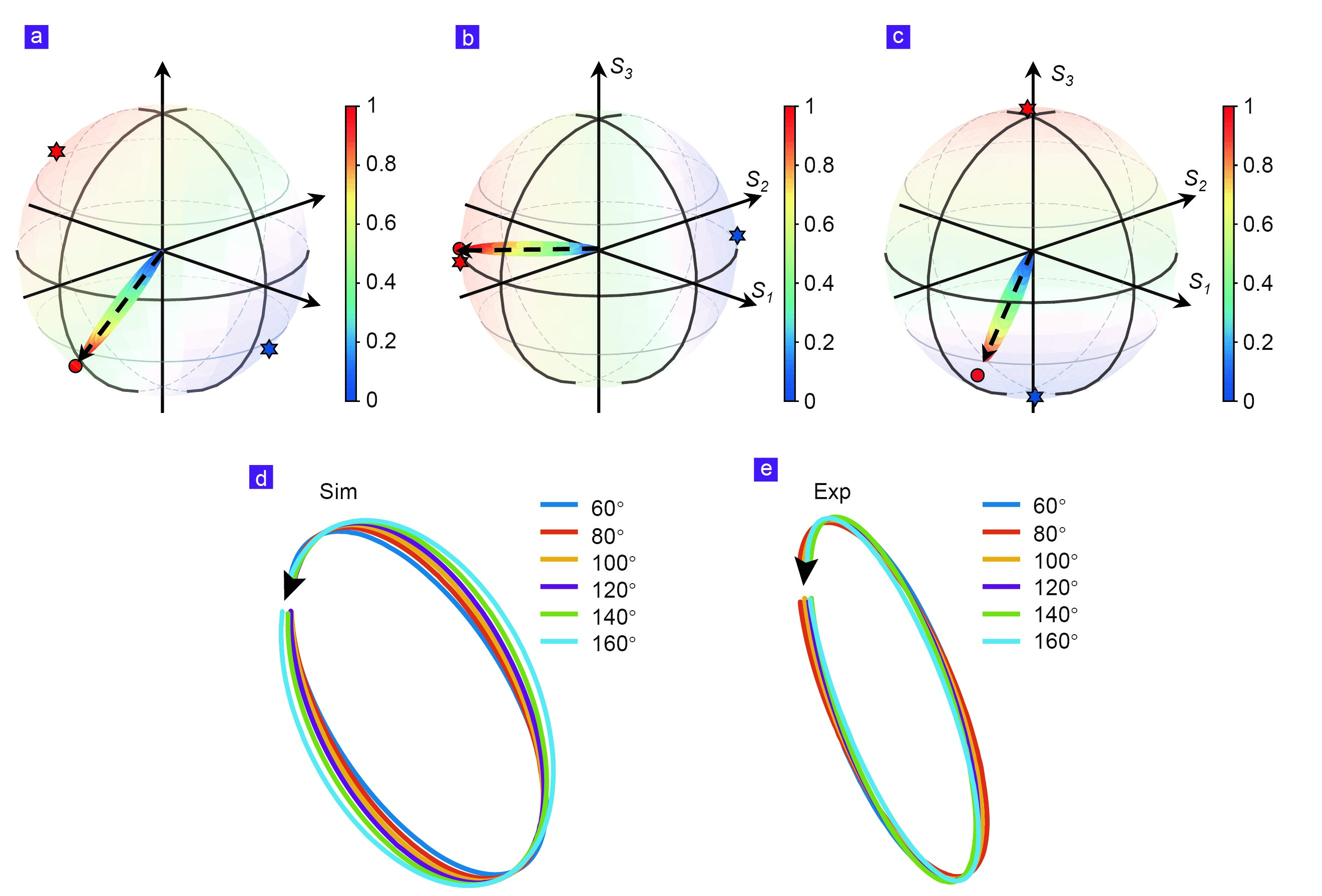


**Figure S3.** **Transmission through APCD metasurface under arbitrary polarization incidences covering the full Poincaré sphere.** The red and blue stars represent the designed polarization incidences with maximum and minimum transmittances through the elliptical (**a**), linear (**b**) and circular (**c**) conversion dichroism metasurfaces. The red dots represent the polarization state of the transmitted beam under incidences with the allowed polarizations by design (red star). The [*S_1_' S_2_ ' S_3_'*] parameters (spindle-like surfaces) illustrate transmitted polarization states under arbitrary polarization incidences and their corresponding transmittances that are indicated by the lengths connecting the points on the spindle surface and the origin. The (d) simulated and (e) experimental output polarization states of the elliptical polarization dichroism metasurface under irradiance by linearly polarized incident beams with different orientation angles.

**Note 3:** **Asymmetric transmission of the APCD metasurface**

Switching the illumination from the frontward to backward directions, the optical response is equivalent to the situation by flipping the metasurface with respect to the axis along the *ψ*-45°direction in the metasurface plane, as shown in Figure S4a. For the designed metasurface illuminated from the backward directions, the Jones matrix can be described as follows:

$\mathbf{J}^{\mathbf{b}} \text{=}\frac{1}{2}\mathbf{R}\left(\psi-45^{\circ}\right)\left(\begin{matrix} e^{-2i\chi} & -1 \\ -1 & e^{2i\chi} \end{matrix}\right)\mathbf{R}\left(45^{\circ}-\psi\right)$ (S24)

the allowed and blocked polarization states of the incident beams from the backward direction are as follows:

$\mathbf{\alpha}^{\mathbf{b}} \text{=}\frac{\sqrt{2}}{2}\mathbf{R}\left(\psi-45^{\circ}\right)\left(\begin{matrix} e^{i\chi} \\ -e^{-i\chi} \end{matrix}\right)=\mathbf{R}\left(\psi-90^{\circ}\right)\left(\begin{matrix} \cos\left(\chi\right) \\ i\sin\left(\chi\right) \end{matrix}\right)$ (S25)

$\mathbf{\beta}^{\mathbf{b}} \text{=}\frac{\sqrt{2}}{2}\mathbf{R}\left(\psi-45^{\circ}\right)\left(\begin{matrix} e^{i\chi} \\ e^{-i\chi} \end{matrix}\right)=\mathbf{R}\left(\psi\right)\left(\begin{matrix} \cos\left(\chi\right) \\ -i\sin\left(\chi\right) \end{matrix}\right)$ (S26)

the allowed and blocked polarization states are switched compared with the case of illumination from the frontward direction. Therefore, the APCD metasurface also manifests arbitrary polarization asymmetry transmission. For the APCD metasurface with parameters (*ψ=*112.5°, *χ*=22.5°), the polarization states and corresponding transmittances being illuminated from the backward direction are shown in Figure S4b. The red and blue stars represent the allowed and blocked polarization states **α^b^***,* **β^b^**, which are identical to polarization states **β** and **α**, respectively. Figure S4c shows the transmission and PD spectra from the backward direction, which preserve the same spectral shapes but with swapped polarization states.


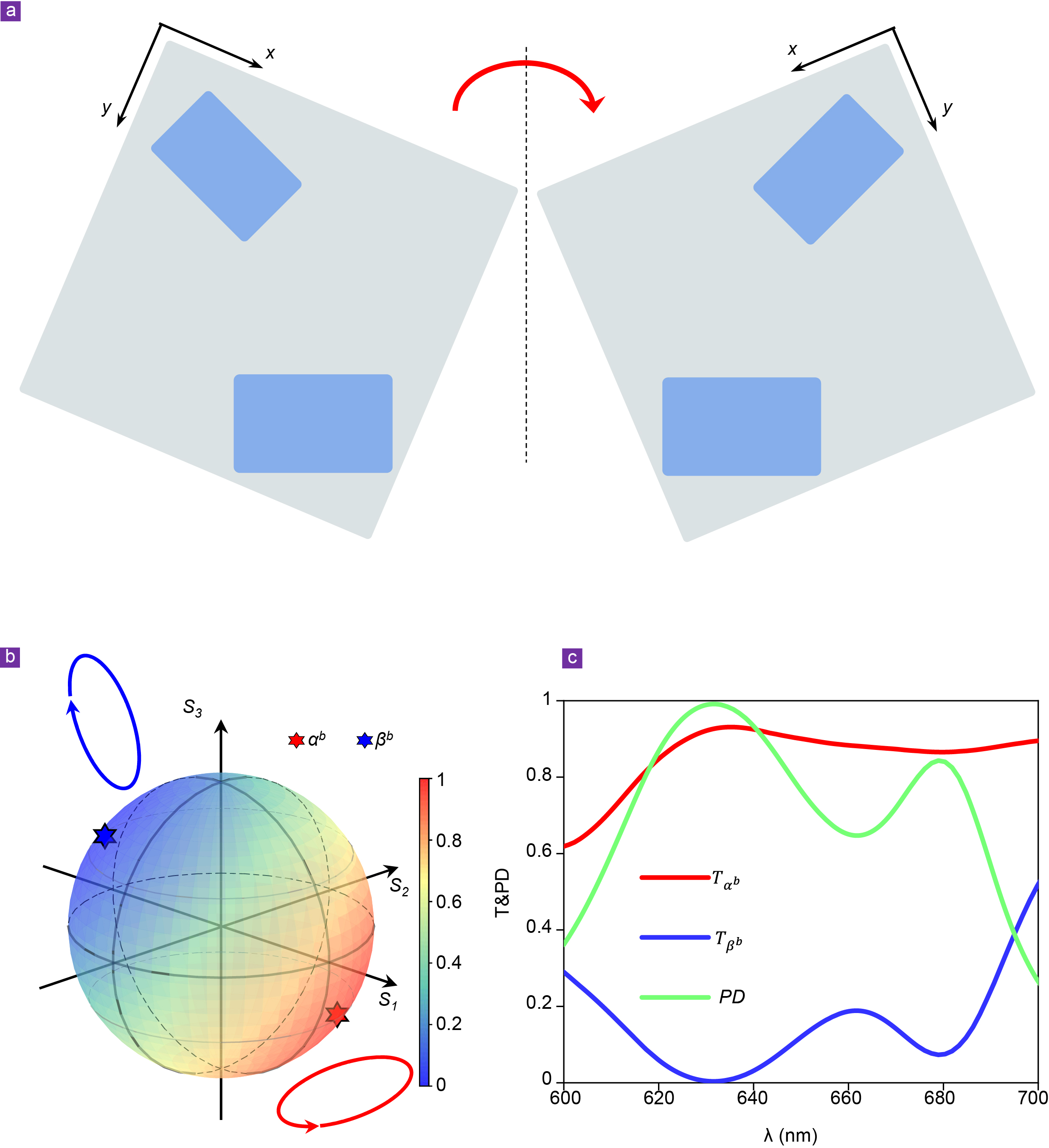


**Figure S4.** **a,** Switching illumination from the frontward to backward directions is equivalent to performing a mirror operation with respect to the axis along the *ψ*-45° direction of the meta-molecule. **b,** The polarization states with maximum and minimum transmittance are marked as the red and blue stars on the surface of the Poincaré sphere. **c,** Transmittances and the PD at broad band wavelength from 600 nm to 700 nm.

**Note 4: Switching of allowed and blocked polarization states in the APCD metasurface**

We can simply change the orientation angle of one of the two terms in Equation S23 to switch the polarization conversion dichroism, which facilitates to construct arbitrary polarizer that can cover the full Poincaré sphere by design with reduced computation load. If we have obtained the optimized parameters for allowing transmission of **α** while blocking **β***,* we can design another APCD metasurfaces that allows transmission of **β** while blocking **α**, or allows transmission of **α*** while blocking **β*** based on the same nanopillars without recalculating.

When we rotate the second nanopillar by 90^o^, the Jones matrix in Equation S23 becomes,

$$\mathbf{J} \text{=}\frac{1}{2}\mathbf{R}\left(\psi-45^{\circ}\right)\left(\begin{matrix} e^{-2i\chi} & 0 \\ 0 & e^{2i\chi} \end{matrix}\right)\mathbf{R}\left(45^{\circ}-\psi\right)+\frac{1}{2}\mathbf{R}\left(\psi+90^{\circ}\right)\left(\begin{matrix} 1 & 0 \\ 0 & -1 \end{matrix}\right)\mathbf{R}\left(-\psi-90^{\circ}\right)$$

$=\frac{1}{2}\mathbf{R}\left(\psi-45^{\circ}\right)\left(\begin{matrix} e^{-2i\chi} & -1 \\ -1 & e^{2i\chi} \end{matrix}\right)\mathbf{R}\left(45^{\circ}-\psi\right)$ (S27)

which represent the case for allowing transmission of **β** while blocking **α**.

The arrangement of meta-molecule is shown in Figure S5a. The orientations of the two nanopillars are *ψ*-45° and *ψ+*90°, respectively, which is equivalent to swapping the length *l* and width *w* of the second nanopillar. The Jones matrix of the elliptical dichroism metasurface for polarization pairs **α** (*ψ* = 112.5°, *χ* = 22.5°) and **β** (*ψ=*22.5^o^*, -χ=-*22.5^o^) becomes as follows:

$\mathbf{J} \text{=}\frac{1}{2}\mathbf{R}\left(67.5^{\circ}\right)\left(\begin{matrix} e^{-2i\chi} & -1 \\ -1 & e^{2i\chi} \end{matrix}\right)\mathbf{R}\left(-67.5^{\circ}\right)$ (S28)

The transmittances of such metasurface illuminated by arbitrary polarization incidences marked in the Poincaré sphere are shown in Figure S5c, which clearly shows that the polarization state **β** can pass through it, while its orthogonal polarization state **α** is blocked. The polarization state of transmitted beams under illumination by state **β** is schematized as the dash blue arrow with flipped handedness. The simulated PD is shown in Figure S5e, which proves high dichroism performance.

Similarly, by rotating the first nanopillar by 90^o^, the Jones matrix becomes,

$$\mathbf{J}=\frac{1}{2}\mathbf{R}\left(\psi+45^{\circ}\right)\left(\begin{matrix} e^{-2i\chi} & 0 \\ 0 & e^{2i\chi} \end{matrix}\right)\mathbf{R}\left(-45^{\circ}-\psi\right)+\frac{1}{2}\mathbf{R}\left(\psi\right)\left(\begin{matrix} 1 & 0 \\ 0 & -1 \end{matrix}\right)\mathbf{R}\left(-\psi\right)$$

$=\frac{1}{2}\mathbf{R}\left(\psi-45^{\circ}\right)\left(\begin{matrix} e^{2i\chi} & 1 \\ 1 & e^{\text{-}2i\chi} \end{matrix}\right)\mathbf{R}\left(45^{\circ}-\psi\right)$ (S29)

which represent the case for allowing transmission of **α*** while blocking **β***.

After rotating the first nanopillar by 90°, the orientation angles of the two nanopillars are *ψ*+45° and *ψ*, respectively, as schematized in Figure S5b. The Jones matrix of the metasurface becomes as follow:

$\mathbf{J} \text{=}\frac{1}{2}\mathbf{R}\left(67.5^{\circ}\right)\left(\begin{matrix} e^{2i\chi} & 1 \\ 1 & e^{-2i\chi} \end{matrix}\right)\mathbf{R}\left(-67.5^{\circ}\right)$ (S30)

The polarization state **α*** will transmit through the metasurface and convert to its handedness-flipped polarization state **α**, while the polarization **β*** is completely rejected as shown in Figure S5d. Figure S5f shows that the PD reaches unity, revealing a perfect dichroism performance. It is worth noting that different arrangements of the nanopillar pair can facilitate arbitrary polarizer design that can cover the full Poincaré sphere by reducing the calculation load for geometry parameter optimization to half Poincaré sphere. In addition, simultaneously rotating the two nanopillars can lead to evolution of allowed and blocked polarization states of **α** and **β**, which is identical to the case of rotating the metasurface by the same rotation angle (Supplementary Note 5).


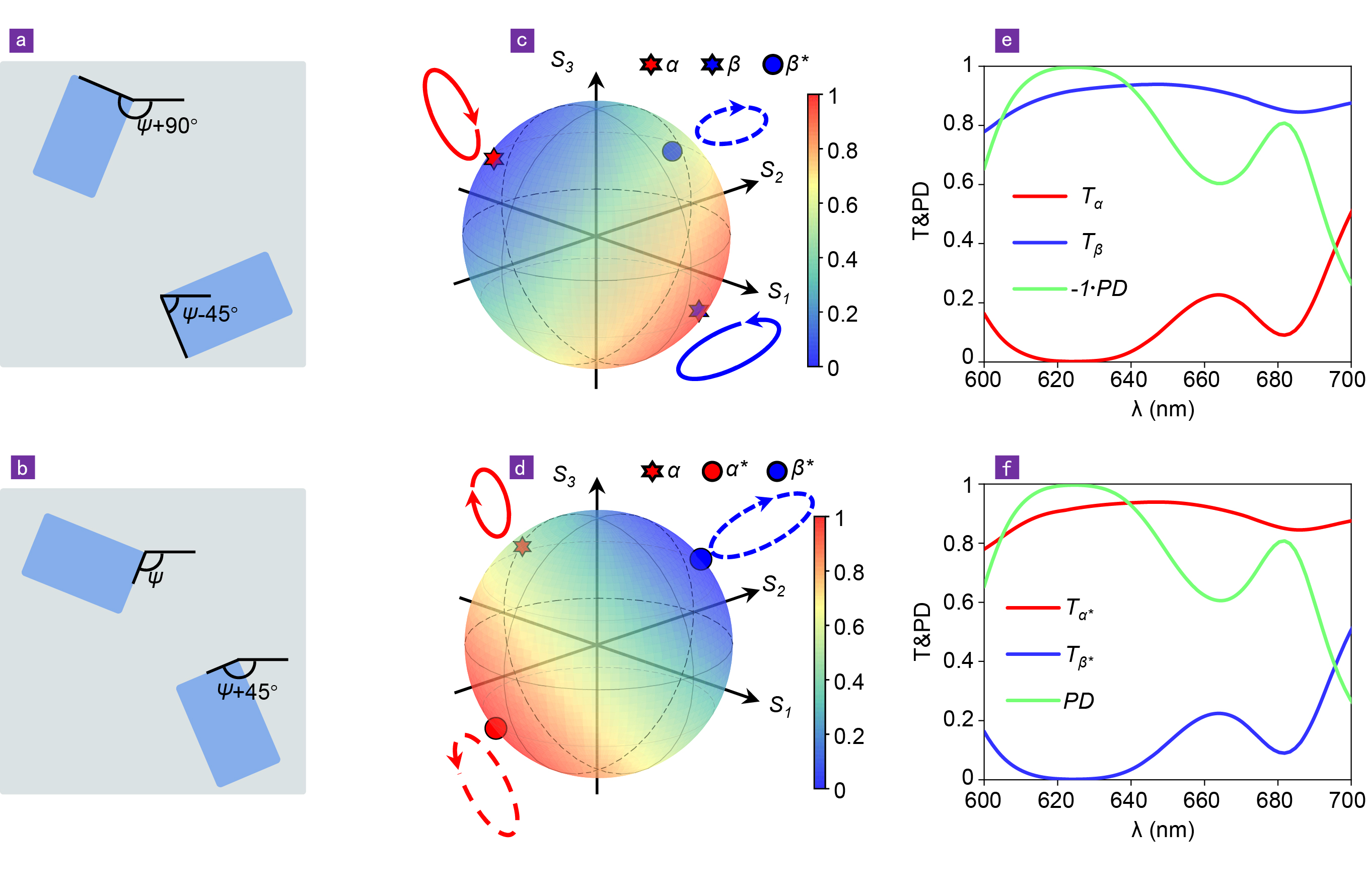


**Figure S5. a**-**b,** Arrangements of the nanopillar pair in different orientation angles. **c**-**d,** Transmittances of metasurfaces shown in **a, b,** respectively, illuminated by arbitrary polarization incidences marked in the Poincaré sphere. **e**-**f,** transmission spectra and the PDs at wavelengths from 600 nm to 700 nm.

**Note 5: The design procedure for arbitrary polarizer covering the full Poincaré sphere**


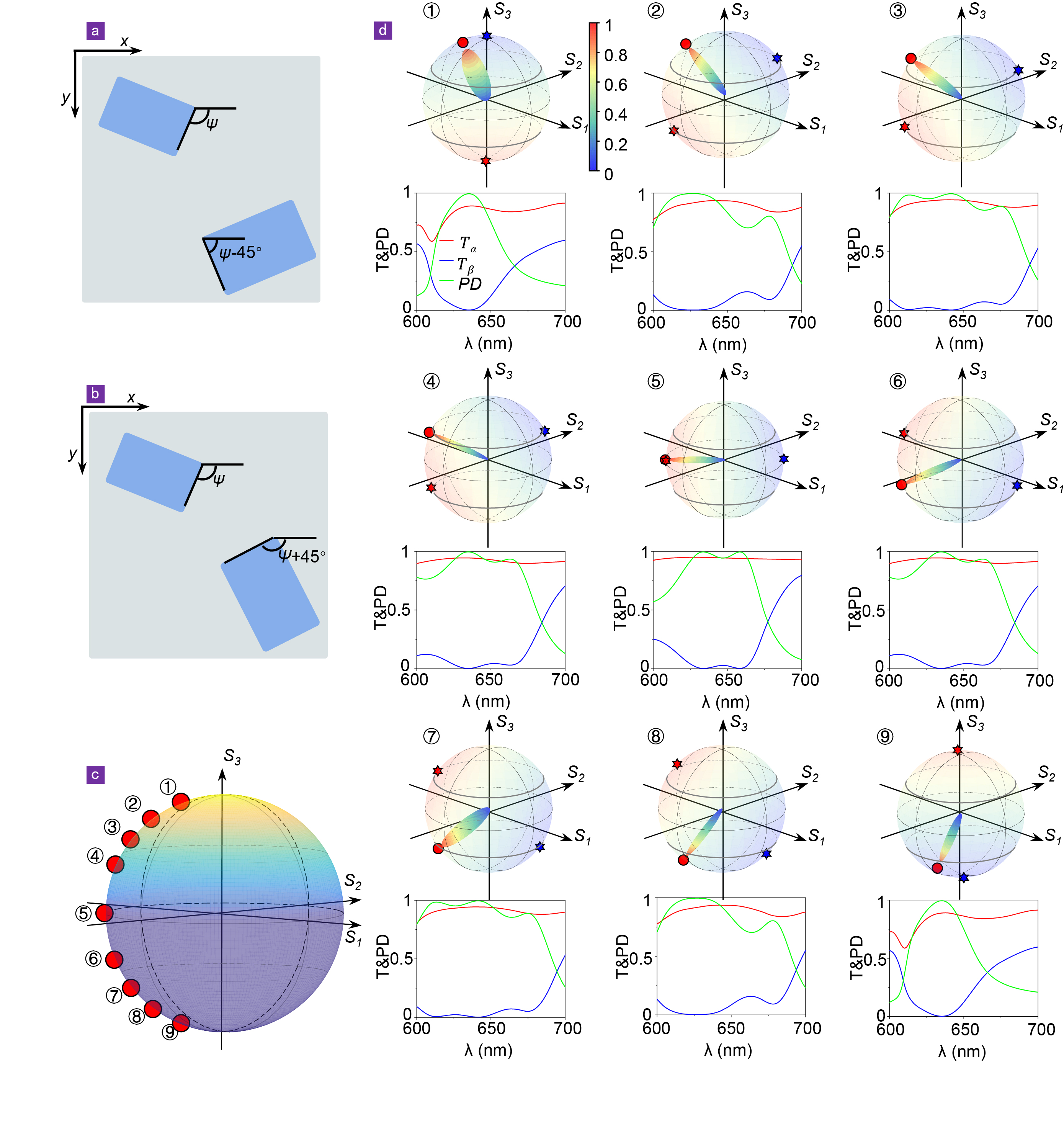


**Figure S6. Designing arbitrary polarizers along the latitude of the Poincaré sphere. a-b,** schematic of the meta-molecules with different orientation arrangements for the upper sphere (①-⑤) and lower sphere polarizers (⑥-⑨), respectively. **c,** Evolution of polarization states ①-⑨ along a latitude on the Poincaré sphere. **d,** Transmittances under arbitrary polarization incidences, and transmission/PD spectra of ①-⑨ polarizers, whose geometric parameters are listed in Table S1. The red and blue stars represent the polarization states with maximum and minimum transmittances, respectively. The red dots represent the transmitted polarization states under incidences with the allowed polarizations by design (red stars).

In the main text, three representative polarizers (elliptical, linear and circular) have been demonstrated based on the APCD metasurface. Here, we introduce the design procedure to obtain arbitrary polarizers with any *ψ* and *χ* values. The procedure begins with polarizations along the latitude of the Poincaré sphere, where the *χ* values change while *ψ* is fixed. Accompanying with the arrangement of rotating the first nanopillar by 90°(switching its length and width) for working on polarization states with reversed ellipticity angle *χ*, we can generate polarization states locating at different positions marked with number from ① to ⑨ on the Poincaré sphere (Figure S6c). The geometric parameters of the nanopillars in those metasurfaces working with nine different ellipticity angles are given in Table S1, and the transmittances and dichroism parameters are plotted in Figure S6d.

The parameter *ψ* of output polarizations can be tailored by simply rotating the metasurface as shown in Figure S7a-b. The transmittances and output polarizations of the elliptical dichroism metasurface working on different orientation angle *θ* of 0, 45°, 90°and 135° are shown in Figure S7b, where the output polarizations evolve along the longitude on the Poincaré sphere as a function of orientation angle *θ* of the metasurface, the trajectory on the Poincaré sphere is shown as the green line, which covers all degrees of the longitude. Therefore, simultaneously rotating the metasurfaces along the longitudinal (with different main axis angles *ψ*) and latitude (with different ellipticity angles *χ*) directions on the Poincaré sphere can generate full Poincaré sphere polarizations (Figure S7c).

**Table S1** The geometry parameter *(l_1_, w_1_ l_2_ w_2_*) of two nanopillars in the metasurface working on nine different ellipticity angles, the unit is nm.

| order | ① | ② | ③ | ④ | ⑤ |
| --- | --- | --- | --- | --- | --- |
| *l_1_, w_1_ l_2_ w_2_* | 75,160,165,100 | 70,130,150,85 | 70,125 ,150,85 | 65, 120,160,70 | 95,95,145,80 |
| order | ⑥ | ⑦ | ⑧ | ⑨ |  |
| *l_1_, w_1_ l_2_ w_2_* | 120,65,160,70 | 125,70,150,85 | 130,70,150,85 | 160,75,165,100 |  |


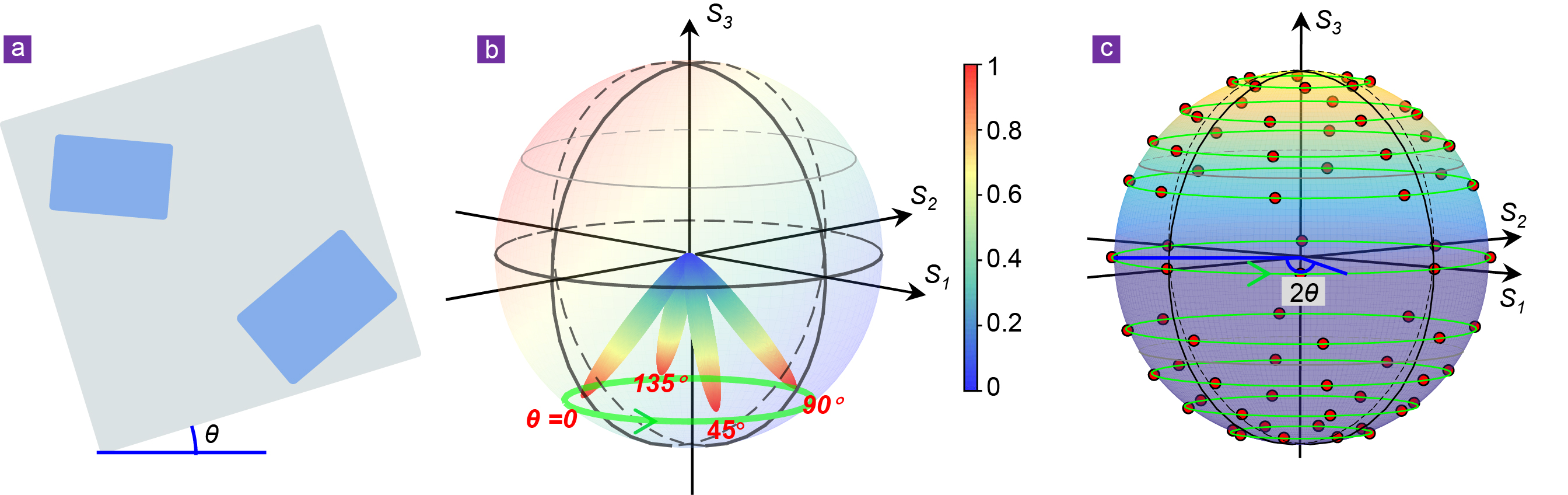


**Figure S7. Designing arbitrary polarizers along longitude lines on the Poincaré sphere.** **a,** schematic of the meta-molecules with a global orientation angle θ. **b,** Transmittances and transmitted polarization states through the elliptical conversion dichroism metasurface with parameters *χ* = 13.5°, by rotating the metasurface with different orientation angles 0^o^, 45^o^, 90^o^, and 135^o^, respectively. **c,** Full Poincaré sphere polarization generation by simultaneously rotating the metasurfaces along the longitudinal (with different main axis angles *ψ*) and latitude (with different ellipticity angles *χ*) directions.

**References:**

1 Wu, L., Tao, J. & Zheng, G. Controlling phase of arbitrary polarizations using both the geometric phase and the propagation phase. *Phys. Rev. B* **97**, 245426 (2018).

2 Menzel, C., Rockstuhl, C. & Lederer, F. Advanced Jones calculus for the classification of periodic metamaterials. *Phys. Rev. A* **82**, 053811 (2010).
